# Cytogenetic Analysis in the Toad Species *Bufo spinosus*, *Bufotes viridis* and *Epidalea calamita* (Anura, Bufonidae) from the Mediterranean Area

**DOI:** 10.3390/genes13081475

**Published:** 2022-08-18

**Authors:** Katerina Guzmán-Markevich, Álvaro S. Roco, Adrián Ruiz-García, Mónica Bullejos

**Affiliations:** Department of Experimental Biology, Faculty of Experimental Sciences, University of Jaén, Campus Las Lagunillas S/N, 23071 Jaén, Spain

**Keywords:** amphibian, bufonidae, chromosome evolution, C-banding, nucleolar organizing region (NOR), FISH, rDNA, interstitial telomeric sequences (ITS), genomic in situ hybridization (GISH)

## Abstract

Taxonomy in Bufonidae witnessed notable transformations. *Bufotes viridis* and *Epidalea calamita*, previously included in genus *Bufo*, were relocated in other genera, while the genus *Bufo* was restricted to members of the earlier *Bufo bufo* group. On the other hand, *Bufo bufo* *sensu lato* now includes four species: *Bufo bufo*, *Bufo spinosus*, *Bufo verrucosissimus* and *Bufo eichwaldi*. In this study, we examined three species of three Bufonidae genera (*B. spinosus*, *B. viridis* and *E. calamita*) by conventional (C-banding and Ag-NOR staining) and molecular (in situ hybridization with probes for telomeric repeats and rDNA loci, and genomic in situ hybridization (GISH)) cytogenetic methods. C-banding patterns are reported for the first time for *B. spinosus* and *E. calamita* populations from Iberian Peninsula and for *B. viridis* from Greece, and reveal several differences with the reported C-banded karyotypes described for other European populations of these species. Silver staining shows size heteromorphisms of the signals at the Nucleolar Organizing Region (NOR). By contrast, FISH with ribosomal probes only reveal size heteromorphism of rDNA sequences in *E. calamita*, suggesting that the differences observed after silver staining in *B. spinosus* and *B. viridis* should be attributed to differences in chromosomal condensation and/or gene activity rather than to differences in the copy number for ribosomal genes. Regarding telomeric repeats, *E. calamita* is the only species with interstitial telomeric sequences (ITS) located on centromeric regions, probably originated by accumulation of telomeric sequences in the centromeric heterochromatin. Finally, we analyzed the composition and distribution of repetitive sequences by genome in situ hybridization. These experiments reveal the accumulation of repetitive sequences in centromeric regions of the three species, although these sequences are not conserved when species from different genera are compared.

## 1. Introduction

Bufonidae is the third largest Anura family (632/630/611 species grouped in 52/53/52 genera, according to [1,2,3] respectively), with a worldwide distribution and extensive presence in the European continent. In the new amphibian taxonomy proposed by Frost [4], the genus *Bufo*, formerly containing a great number of amphibian species, was reorganized. Most species were moved to other genera and the genus *Bufo* was restricted to members of the earlier *B. bufo* group. In this way, *B. viridis* and *E. calamita*, previously included in the genus *Bufo*, were relocated to other genera [4,5,6], although not without controversy [7,8].

The taxonomy of these groups is far from being solved, and new changes are proposed as more information is obtained from different populations [6,9,10]. For example, the genetic differentiation observed between populations of the *B. bufo* group precluded the review of their taxonomy recognizing additional species [11]. Thus, according to mitochondrial and nuclear markers, *B. bufo* populations from Iberian Peninsula were included in a different species, *B. spinosus* [12,13,14]. *B. bufo* is found in most of Europe (from northern and eastern France into Russia, including toads from Great Britain, Scandinavia, Italy, the Balkans and the larger part of Turkey), while *B. spinosus* is distributed in North Africa, Iberia and western France. Contact areas of these lineages are very blurred, since populations of *B. spinosus* have been found in British Isles Jersey [15] and in Tunisia [16].

The species *B. bufo*, *B. spinosus*, *E. calamita* and *B. viridis* have a conserved karyotype with 2n = 22 (fundamental number (FN) = 44), including six pairs of relatively large and five pairs of distinctly smaller chromosomes (they represent about 80% and 20% of total genome, respectively [17], and their relative lengths range between 9.4–16.2 and 2.9–4.6, respectively [18]). For each chromosome pair, its relative size in *B. bufo sensu lato* is longer than in *B. viridis* and *E. calamita* [19]. On the other hand, the differences between these two latter species are only observed in large chromosomes, with those of *B. viridis* being longer than in *E. calamita* [19]. These differences are not due to differences in the amount of heterochromatin, since these species show similar number of C- and Q-bands, with constitutive heterochromatin predominantly located at both centromeric and telomeric regions [20].

The wide distribution of *B. bufo sensu lato*, *B. viridis* and *E. calamita* results in large variation of cytogenetic patterns between populations ([17] and references therein). Most karyotypic and cytogenetic studies performed in these three species have used Central European specimens [17,20,21,22]. However, detailed cytogenetic analysis of *B. spinosus* and *E. calamita* from the Iberian Peninsula and *B. viridis* from Crete (Greece) are not available.

In this work we have characterised *B. spinosus* and *E. calamita* specimens from Iberian Peninsula and *B. viridis* from Greece, using conventional (C-banding and Ag-NOR staining) and molecular (in situ hybridization with probes for telomeric repeats and rDNA loci, and genomic in situ hybridization (GISH)) cytogenetic methods. The three species were compared, among them and with the data available from Central Europe specimens of the same species [17,20,22], providing information about the genomic/chromosomal changes that occurred during the evolution of this group. We have observed new undescribed interstitial C-bands, revealing population variability regarding heterochromatin distribution. Additionally, the three species present accumulation of different repetitive sequences in their centromeric regions. This is especially evident in *E. calamita*, with centromeric ITSs located on all chromosomes. The data gathered offer new chromosomal markers that could contribute to the taxonomic classification of bufonids. Finally, independently of the classification of *B. bufo* from Iberian Peninsula as *B. spinosus* species or *B. bufo spinosus* subspecies [12,14,23], the results reported here provide the first detailed cytogenetic analysis of this taxonomic group.

## 2. Materials and Methods

### 2.1. Animals Analyzed

The geographic origin of the samples, their ploidy level, sex, developmental stage (adult or tadpole), number of animals used and source of metaphase chromosomes (bone marrow or cell culture) are indicated in Table 1. *B. bufo* and *B. spinosus* samples were confirmed by sequencing their 16S rDNA [24]. The sex of the tadpoles was stablished after sectioning their paraffin-embedded gonads (ovaries can be differentiated by the presence of the ovarian cavity).

Animals were collected in accordance with applicable regulations for the protection of terrestrial wild animals. Capture permits for *B. spinosus* and *E. calamita* were provided by the Junta de Andalucía, Dirección General de Gestión del Medio Natural (2010, 2012). *B. viridis* clutches were sampled for a previous work [25]. *B. bufo* samples (DNA and mitotic chromosomes) were a generous gift from Francesco Zaccanti’s laboratory. Mitotic chromosomes from an adult *E. calamita* were available from a previous work [25]. All animal protocols were approved by the Ethics Committee for Research on Animals of the University of Jaén (2009). The care and sacrifice of animals used in this research was conducted in accordance with policies on animal care provided by Spanish and EU regulations. When applicable, this study is reported according to the ARRIVE guidelines.

### 2.2. Cell Culture and Chromosome Preparations

To reduce the impact of our research on the amphibian populations used in this work, our study mainly uses chromosomes obtained from secondary cell cultures derived from larvae tissues, avoiding the sacrifice of adult individuals from populations that may be in regression [26,27].

Tadpoles were euthanized by immersion in buffered 2% Tricaine methanesulfonate (MS 222; Sigma-Aldrich, Darmstadt, Germany) and then decapitated and dissected. Primary cell cultures were prepared from tadpole limbs as described in [28]. Secondary cell cultures were maintained in Dulbecco’s modified Eagle’s medium (DMEM) (Sigma-Aldrich, Taufkirchen, Germany) supplemented with 10% foetal bovine serum (PAA Laboratories, Cölbe, Germany), and 100 µg/mL penicillin, 100 U/mL streptomycin and 2.5 µg/mL amphotericin B (all antibiotics from Sigma-Aldrich, Darmstadt, Germany). Cell cultures were maintained at 28 °C in a humidified atmosphere of 5% CO_2_ in air. When cells were semiconfluent, colcemide (Karyomax, GIBCO, Gaithersburg, MD, USA) was added at final concentration of 100 ng/mL for 5 h. Cells were collected after trypsinization and incubated in 0.56% KCl for 20 min at room temperature. After hypotonic treatment the cells were centrifuged at 1000 rpm for 10 min and fixed in ice-cold methanol:acetic acid (3:1) as described in [20]. Samples were stored at −20 °C in fixative until use.

Mitotic chromosomes from adult individuals (*B. bufo* and sample M4 from *E. calamita*) were obtained from bone marrow after a 2 h in vivo colchicine treatment (2%) according to [29].

### 2.3. Chromosome Banding

C-banding was performed as described by [30] with minor modifications. Briefly, slides were incubated in 0.15N HCl for 20 min at room temperature, in a saturated Ba(OH)_2_ solution for 20 min at 50 °C, and then in 2 × SSC for 45 min at 60 °C. Finally, slides were rinsed in distilled water and stained with 10% Giemsa for 30 min.

The nucleolus organizer regions (NOR) were labelled with AgNO_3_ following the protocol described by [31]. Briefly, 40 µL of freshly prepared silver nitrate buffer (0.1 g AgNO_3_ in 100 µL of formic acid solution (a few drops of formic acid in 200 mL of H_2_O)) were applied to each preparation, covered with a coverslip and incubated in a humidified chamber at 60 °C. When a brownish colour developed, the slides were washed with distilled water and air-dried.

### 2.4. In Situ Hybridization: FISH and GISH

Probes were biotin-labelled for further use in FISH (rDNA and telomeric repeats) and GISH (genomic DNA) experiments. Telomeric repeats, (TTAGGG)_n_, were synthetized and labelled by PCR as described in [28]. One μg of recombinant plasmid (pDmr.a51) with a 11.5 kb insert encoding 18S and 28S ribosomal units from *Drosophila melanogaster* [32], was labelled using the Nick Translation Mix (Sigma-Aldrich, Darmstadt, Germany) according to the manufacturer’s instructions. Genomic DNAs from *B. bufo*, *B. spinosus*, *B. viridis* and *E. calamita* (1 μg) were also labelled by nick translation using the same protocol. Probes were ethanol-precipitated with salmon sperm DNA and ammonium acetate 5M, resuspended in 120 μL of 50% formamide/2 × SSC, denatured for 6 min at 75 °C and chilled in ice prior hybridization.

Slides were incubated with RNase A (Roche, Mannheim, Germany) solution (100 μg/mL in 2 × SSC) for 1 h at 37 °C, washed three times in 2 × SSC for 5 min each, and then incubated in Pepsin (Applichem, Darmstadt, Germany) solution (50 µg/mL in 0.01 N HCl) at 37 °C for 5 min. After two washes in 2 × SSC for 5 min, the slides were fixed in 1% formaldehyde (Applichem, Darmstadt, Germany) (*v*/*v*) in PBS for 10 min and washed 3 times for 5 min each in 2 × SCC.

Pre-treated slides were dehydrated in an ethanol series (70, 90 and 100%, 5 min each) before air drying. Chromosomes were denatured at 70 °C for 2 min in 70% formamide/2 × SSC and again dehydrated in an ethanol series before air drying.

Hybridization was performed overnight at 37 °C in a humidified chamber using 10–15 μL of resuspended probe. Post-hybridization washes included 3 washes with 50% formamide/2 × SSC at 37 °C for 5 min and 2 washes with 2 × SSC for 5 min. After a blocking step with 4 × SSC/5% blocking reagent (Roche, Mannheim, Germany) in a humidified chamber for 1 h at room temperature, the slides were incubated in 4 × SSC/5% blocking reagent containing avidin-FITC (Vector Laboratories, Burlingame, CA, USA) for 1 h. The signal was enhanced using a modified avidin-FITC/biotinylated anti-avidin system (Vector Laboratories, Burlingame, CA, USA).

The FITC signal enhancement was achieved by three applications of the primary (avidin-FITC) and two applications of the secondary antibody (biotinylated anti-avidin) solutions at 37 °C for 30 min each (four and three applications, respectively, were used for telomeric repeats), with washes in 4 × SSC/0.05% Tween 20 in-between the applications of the antibody solutions. Finally, the slides were washed with PBS (4 times) and mounted with Vectashield DAPI anti-fade medium (Vector Laboratories, Burlingame, CA, USA). More than five metaphase plates were analyzed for each combination of species and probe.

### 2.5. Microscopy and Image Capture

Slides were examined using an Olympus BX-51 fluorescence microscope. Separate images from each filter set were captured using a cooled CCD camera (OLYMPUS DP70, Olympus Optical Co., Ltd., Tokyo, Japan). Images were optimized for best contrast and brightness using Adobe Photoshop CC software v14.0 (Adobe Systems Incorporated, San Jose, CA, USA), employing only the functions that are applied equally to all pixels in the image. In some occasions, distant chromosomes were brought closer together.

### 2.6. Chromosome Measurements

To measure the chromosome lengths, we used 97 Ag-NOR stained metaphases from three individuals (cultures). Specifically, 12 from male T8, 18 from female T10 and 67 from female T12. Total length and chromosome arm’s length were measured in both chromosomes from pair 6 using ImageJ v1.52a [33] (Available online: http://imagej.nih.gov/ij/ (accessed on 5 July 2018)). Pair 6 was easily identified, as it harbors the NOR in this species. The relative length of each chromosome 6 was calculated as a ratio of chromosome length to the total length of pair 6 (in %). The one-way analysis of variance test (ANOVA) was performed by SPSS 27.0 (SPSS, Armonk, NY: IBM Corp) to determine statistically significant differences between the means of males and females.

## 3. Results

### 3.1. C-Banding

The specimens from *B. spinosus*, *B. viridis* and *E. calamita* analyzed showed conserved karyotypes with 2n = 22. All chromosomes in the karyotype are metacentric or submetacentric (except for the subtelocentric pair 11 in *E. calamita*), and they can be grouped in six pairs of relatively large and five pairs of relatively small chromosomes. The chromosome pairs were numbered as in [19,20].

**Figure 1:**
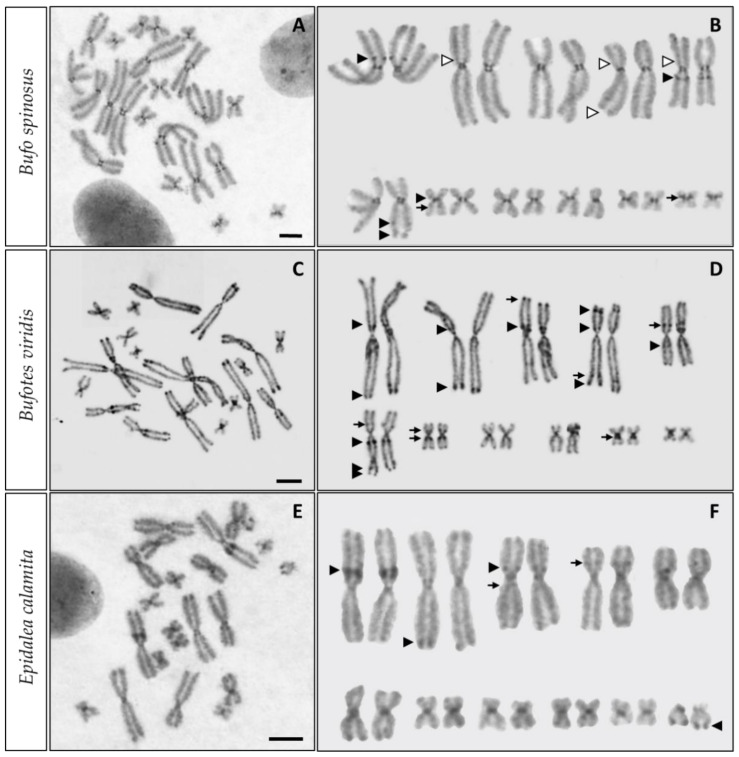
C-banded metaphase chromosomes from *B. spinosus* (**A**), *B. viridis* (**C**) and *E. calamita* (**E**). For each species, a C-banded metaphase (**A**,**C**,**E**) and the corresponding karyotype (**B**,**D**,**F**) are included. C-positive bands already described in other populations are indicated by black arrow heads, while those non-described previously are depicted by black arrows. White arrow heads depict previously described C-positive bands not identified in our samples. C-bands in *B. spinosus* were compared with those published for *B. bufo* [17,20,21,34]. C-bands in *B. viridis* were compared with data from [17,20,22,35], while C- banding information in *E. calamita* was obtained from [20]. Scale bar: 2.5 μm.

**Figure 2:**
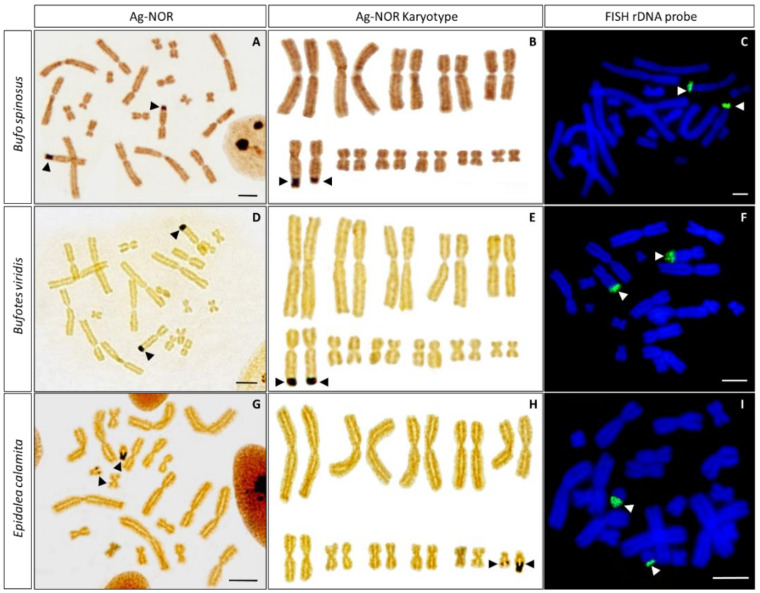
Silver staining and FISH with rDNA on metaphase chromosomes from *B. spinosus* (**A**–**C**), *B. viridis* (**D**–**F**) and *E. calamita* (**G**–**I**). For each species, a silver stained metaphase (**A**,**D**,**G**) with the corresponding karyotype (**B**,**E**,**H**) and fluorescent in situ hybridization (FISH) with rDNA (18 + 28S) from *D. melanogaster* (**C**,**F**,**I**) are included. For each species, Ag-NOR and FISH are from the same individual. The black arrow heads point to the NORs while the FISH signals are pointed out by the white arrow heads. Scale bar: 2.5 μm.

C-positive bands were observed in the centromeric position of all chromosomes, although they were more evident in *B. spinosus* (Figure 1A,B). Interstitial heterochromatic bands were also observed in all examined species. In *B. spinosus* intense C-positive interstitial bands were located on the short arm of chromosome 1 (1p) and long arm of chromosome 5 (5q), with both bands positioned close to the centromere. Less intense interstitial bands at telomeric and subtelomeric positions of chromsome 6 (6q), delimitate the secondary constriction where the NOR is located in this species (Figure 2B). Other C-positive bands not described previously in *B. bufo* are located on 7q and 11p, both close to the centromere.

C-positive interstitial bands in *B. viridis* (Figure 1C,D) were observed in the short arm of chromosomes 1 (1p), 2 (2p), 3 (3p) and 4 (4p), and in the long arm of chromosomes 5 (5q) and 6 (6q), close to the centromere in all cases. Additionally, telomeric bands were also observed in 1q, 2q and 4q. As described in *B. spinosus*, two heterochromatic bands delimit the secondary constriction where the NOR is located (subterminal position of chromosome 6, see Figure 2E). Non-described C-bands were identified in chromosomes 3p (terminal), 4q (subterminal interstitial band), 5p (pericentromeric), 6p (pericentromeric), chromosome 7 (pericentromeric heterochromatic bands in 7p and 7q), and in chromosome 10q (pericentromeric).

In *E. calamita* the positive interstitial bands were observed close to the centromere of the short arms of chromosomes 1 (1p) and 3 (3p) (Figure 1E,F). Intense signals are also observed at the distal position of the long arm of chromosome 2 (2q) and chromosome 11 (11q) (location of the secondary constriction corresponding to the NOR, see Figure 2H). Non-described C-bands were identified in 3q and 4p (pericentromeric in both cases), while the telomeric bands described in [20] were not observed.

Faint C-positive staining for telomeric heterochromatin was observed in some large chromosomes of all species and in some small chromosome pairs of *B. viridis*. C-positive telomeric signals were not evident in the small chromosome pairs of *B. spinosus* and *E. calamita*, although they can be observed in some metaphases.

### 3.2. Ag-NOR Staining

*B. spinosus*, *B. viridis* and *E. calamita* show positive silver staining only in one chromosome pair, where the secondary constriction corresponding to the NOR is located (Figure 2). Positive signals in *B. spinosus* and *B. viridis* are located at the distal end of the long arm of chromosome 6 (6q) (Figure 2A,B and Figure 2D,F, respectively), while in *E. calamita* it is observed at terminal position of the long arm of the smallest chromosome pair (11q) (Figure 2G,H).

When NOR-signals were present, they were located in both homolog chromosomes. NOR size polymorphisms between individuals and from homologue to homologue were observed in all species analyzed, although differences were less evident in *B. viridis* than in *B. spinosus* or *E. calamita* (Figure 2 and Table 1).

**Figure 3:**
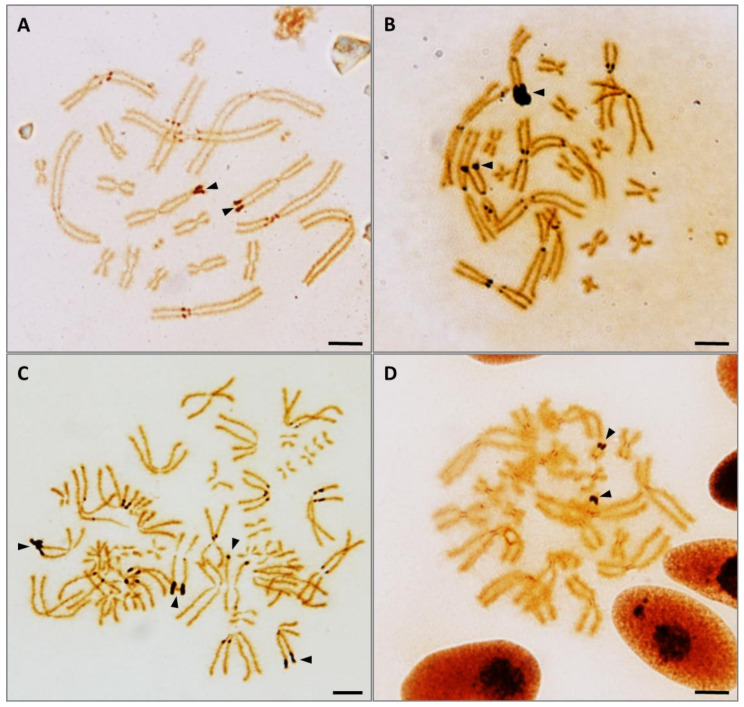
Silver staining of metaphases from *B. spinosus* (**A**–**C**) and *E. calamita* (**D**). Positive signals are observed at distal ends of the long arm of chromosome pair 6 in *B. spinosus*, or chromosome pair 11 in *E. calamita* (arrow heads). Occasionally, intense signals can be also observed on the pericentromeric regions of large pairs in *B. spinosus* (**A**–**C**). Additionally, at times, less evident signals can be observed in centromeric regions of all chromosome pairs in *E. calamita*. Scale bar: 2.5 μm.

Occasionally, positive silver staining was also observed at positions different from the NOR in some metaphase spreads from *B. spinosus*. These signals are located at the centromeres of large chromosomes, usually in the form of two symmetric signals located at one or both sides of the centromere of poorly condensed chromosomes (Figure 3A–C). Additionally, at times, less intense positive silver staining was observed at the centromeres of *E. calamita* (Figure 3D).

It has been proposed that *B. spinosus* has an XX/XY sex chromosome system with pair 6 being the sex chromosome pair [18]. This conclusion is based on the differences in the length of chromosome 6 when males (two individuals, three and ten metaphase plates) and females (one individual, three metaphase plates) are compared. To check if this feature is widely spread, we have measured the length (each arm and complete) of both chromosomes from pair 6 in our samples (two females and one male) using a higher number of metaphase plates (12, 18 and 67 metaphases for samples T8, T10 and T12, respectively) (Table 2). The analysis of one-way ANOVA indicates there are no significant differences between samples (*p*-value of 0.287, 0.304 and 0.791 for the differences in lengths for chromosome 6p, 6q and 6, respectively). According to our results, the length measurements of chromosome 6 are not valid to establish this chromosome as the sex chromosome pair in *B. spinosus* [18].

### 3.3. In Situ Hybridization with Ribosomal DNA

We implemented FISH with a 18S + 28S rDNA probe from *D. melanogaster* to check whether the observed size polymorphisms for the NOR in these samples were due to differential activation of ribosomal genes or to differences in the copy number of ribosomal cistrons. The hybridization signal obtained after FISH with this probe is present only in one chromosome pair in each species, at the position where the secondary constriction and the NOR signals have been identified (Figure 2C,F,I). Size polymorphisms for FISH signals were clear in *E. calamita*, but not so evident in *B. spinosus* or *B. viridis*. No other positive signals were observed in any metaphase analyzed from *B. spinosus*, *B. viridis* or *E. calamita*.

### 3.4. In Situ Hybridization with Telomeric Repeats

**Figure 4:**
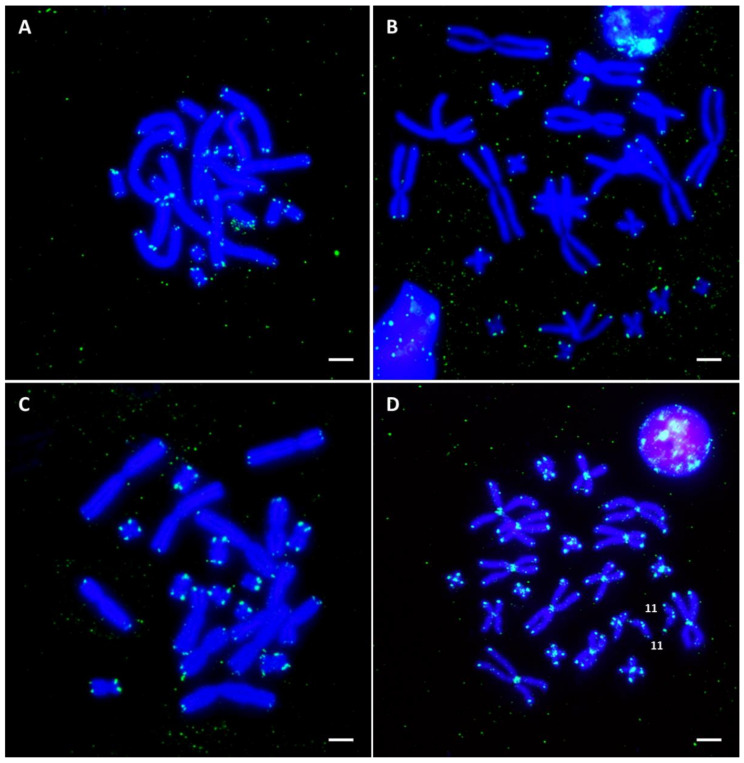
In situ hybridization using a telomeric probe, labeled with biotin 11–dUTP (after four rounds of amplification), on metaphase chromosomes from *B. bufo* (**A**), *B. spinosus* (**B**), *B. viridis* (**C**) and *E. calamita* (**D**). Scale bar: 2.5 μm.

The signal of the FISH with telomeric repeats was located, as expected, on the terminal regions of all chromosomes in the analyzed species (Figure 4). In *B. viridis*, the intensity of the signal was higher on the five smaller pairs compared to the six longer ones (Figure 4C). In addition, blocks of telomeric repeats located in non-terminal regions of the chromosomes were identified in *E. calamita* (Figure S1F). These interstitial telomeric sequences (ITSs) are located in the centromeric regions of all chromosomes (not evident in the subtelocentric pair 11, see Figure S1). To discard the possibility these ITSs were produced as consequence of chromosomal reorganizations that occurred during cell culture, we also performed FISH with a telomeric probe on chromosomes from an adult *E. calamita* male (Figure 4D). No differences in the distribution of the telomeric signal were observed between chromosome samples from tadpoles (cell culture) or from adults (bone marrow) (Figure S1).

### 3.5. In Situ Hybridization with Genomic DNA (GISH)

Anurans have a large number of medium-repeated sequences that can be missed by classical cytogenetic techniques, such as C-banding. To obtain information on the amount and distribution of repeated DNA sequences, FISH experiments have been performed on metaphase chromosomes of these species using genomic DNA as a probe (genome in situ hybridization or GISH). In this way, it is possible to obtain information on the amount and distribution of repeated sequences in each species, as well as on the similarities between repetitive DNAs of related species.

**Figure 5:**
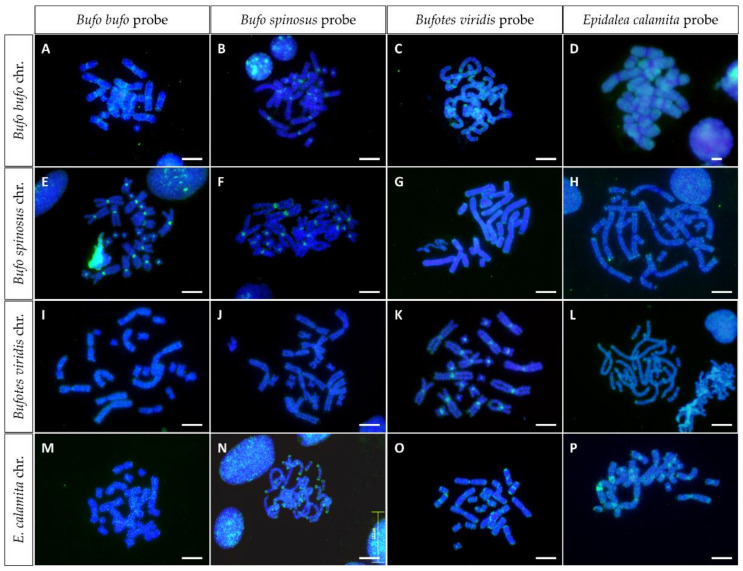
In situ hybridization using genomic DNA (GISH) from *B. bufo* (**A**,**E**,**I**,**M**), *B. spinosus* (**B**,**F**,**J**,**N**), *B. viridis* (**C**,**G**,**K**,**O**) and *E. calamita* (**D**,**H**,**L**,**P**) as probe, on metaphase chromosomes from *B. bufo* (**A**–**D**), *B. spinosus* (**E**–**H**), *B. viridis* (**I**–**L**) and *E. calamita* (**M**–**P**). The probes were labeled with biotin 11–dUTP and results were obtained after three rounds of immunological amplification using an avidin-FITC/biotinylated anti-avidin system. Scale bar: 5 μm.

The results of the hybridization of the chromosomes from *B. bufo*, *B. spinosus*, *B. viridis* and *E. calamita* with their own genomic DNA (GISH) are shown in Figure 5A,F,K,P. The hybridization pattern shows that *B. bufo*, *B. spinosus* and *B. viridis* have a large accumulation of repetitive DNA in the centromeres of all chromosomes, while in *E. calamita* the signal spreads along chromosome arms, with accumulation at the centromeres and at the tip of the chromosomes. Of note is the presence in *E. calamita* of intense signals in 1p and 3p in pericentromeric position (see Figure S2), probably due to the presence of satellite DNA BamHI-800 in this position [25].

The conservation of the repetitive sequences identified by GISH was addressed by cross-GISH (Figure 5). The accumulation of repetitive DNA in centromeric regions of all chromosomes when each species is hybridized with its own genomic DNA is only evident when *B. bufo* and *B. spinosus* are cross hybridized (Figure 5B,E). When chromosomes and genomic DNA are from different genera (*Bufo*, *Bufotes* or *Epidalea*), the centromeric intense signals are no longer observed, although disperse signals are observed along chromosomal arms. Interesting hybridization patterns are observed when genomic DNA from *E. calamita* is used as probe on *B. bufo* and *B. spinosus* chromosomes (Figure 5G,H), showing absence of hybridization signal in the centromeric regions. Similar hybridization pattern, although less evident, is observed when *B. viridis* genomic DNA is used as probe with the chromosomes of the previous species (Figure 5C,G). Finally, the ends of *E. calamita* chromosomes show intense hybridization signals with *B. spinosus* probe (Figure 5N), while the probe from *B. viridis* reveals less intense signals in telomeric and in some centromeric regions that could be due to BamHI-800 satellite DNA [25] (Figure 5O).

## 4. Discussion

The C-banded karyotypes of the species analyzed in this work did not show big differences with those of previously studied populations [17,20,21,34], although several new and missing bands have been identified in these species (see Figure 1).

According to the current taxonomy, *B. spinosus* (Daudin, 1803), together with *B. bufo* (Linnaeus, 1758), *Bufo verrucosissimus* (Pallas, 1814) and *Bufo eichwaldi* [11], is part of the *B. bufo* species group [12,15]. The karyotypes of these species have been studied previously [17,19,20,21,34,36,37,38], although only a Giemsa-stained karyotype is available for *B. spinosus* [18]. According to our results, in addition to the centromeric heterochromatic bands, *B. spinosus* metaphases show several C-positive interstitial bands already described in European [20], Yugoslavian [17] and Russian [21,34] populations of *B. bufo sensu lato*. In our samples, we have not been able to identify some pericentromeric and telomeric bands previously described for Eurasian (2p, 4q), or Russian (4p, 5p) samples [20,34]. On the other hand, C-banding in *B. spinosus* also reveal C-positive bands not described previously in *B. bufo* samples: a proximal band on 7q, and a pericentromeric band on 11p (also suggested for *B. bufo* from Russia by [34]). The observed differences in the C-banding patterns between *B. spinosus* and *B. bufo* may reveal species differences, although population variations could not be discarded. More samples from different geographical origins should be analyzed before a conclusion could be reached. In any case, the karyotype from *B. spinosus* from Figure 1 is the first available C-banded karyotype for this species.

The variations described in interstitial bands from *B. viridis* and *E. calamita* may be due to methodological differences, as we have used chromosome preparations obtained from cell cultures, a procedure that can provide less condensed chromosomes. However, they can also indicate the existence of population differences in heterochromatin distribution, something that would not be surprising in species with such a wide range of distribution throughout the European continent. Differences in karyotypes and C-banding patterns between species of the Bufonidae family have been described, and reveal intraspecific chromosomal variations [17,20,21,34]. The relationship between these differences and speciation events, if any, needs further analysis.

Marker C-positive bands have been used to distinguish between toad species (e.g., distinguish different groups of *Bufo japonicus* [39]). However, according to our results, C bands show variation between populations, and may not be applicable to distinguish between close related species. This is the case of the intense C-positive interstitial band located on 5q in *B. bufo* (and in *B. spinosus*, this work), proposed as cytogenetic marker to differentiate between *B. bufo* (band present) and *B. viridis* (band absent) [17]. However, our results show that this marker band is also present in some populations of *B. viridis*.

Our results with Ag-NOR staining agree with the karyotypes reported previously for these species [17,18,20,22,40,41]. Occasionally, Ag-positive signals are also observed at positions other than the NOR (Figure 3). These signals are not due to the presence of ribosomal cistrons in locations other than the NOR, since no FISH signal is observed on these locations when 18 + 28S rDNA is used as probe (Figure 2). Similar centromeric Ag-positive signals, different from those of active NORs, has been observed in bufonids (e.g., *Bufo paracnemis* [42]) and in other species [43,44,45]. It has been proposed that they can be generated by the binding of silver nitrate to acidic (non-histone) proteins (similar to those that can be found in the NOR during its active transcriptional phase) located in centromeric or pericentromeric regions. An alternative explanation proposes that they originate from de-condensation of heterochromatin [44], exposing centromeric and the surrounding pericentromeric heterochromatin to acidic proteins.

NOR signals were commonly evident on both homologues and a size heteromorphism was observed with silver staining in all species analyzed. This is quite common in amphibians and had been described before in species of the family Bufonidae [20,40,46]. The frequency of heteromorphic NORs was higher in samples from *B. spinosus* and *E. calamita*, while homomorphic NOR signals are more frequent in *B. viridis*. In contrast, size polymorphisms for the NOR revealed by FISH are only evident in *E. calamita*. These results indicate that the size polymorphisms observed by Ag-staining are not always attributable to differences in copy number for rDNA genes, since NOR size polymorphisms do not correlate with differences in FISH signal in *B. spinosus*. In this species, the differences observed with silver staining could be due to differences in activity between both homologues. On the other hand, the differences observed in *E. calamita* could be attributed to differences in the number of rDNA genes, as shown by FISH, but also to differences in accessibility of these regions to acidic proteins [40].

Based on differences between chromosome 6 homologues in *B. spinosus* males but not in females, [18] proposed that this species has a XX/XY sex chromosome system. Our results do not support such conclusion, as the differences we observed between both chromosome 6 homologues (or between their p or q arms) are not significant when male and female samples are compared (Table 2). The difference between our results and those from [18] could be attributed to differences between populations, but also to differences in the number of metaphase plates analyzed. Of note is the high variability observed in the size of the chromosomes of different metaphases from a given sample.

According to the Animal Genome Size Data-base [47] (Release 2.0), the DNA content per nucleus in *B. bufo sensu lato* varies between 5.82 and 7.75 pg, whereas in *B. viridis* and *E. calamita* this value ranges between 3.82–6.84 pg and 4.01–5.70 pg, respectively (there is no specific information available for *B. spinosus*). Considering that the C- or Q-banded karyotypes do not show large heterochromatin blocks in these species (Figure 1 and [20], respectively), it is unlikely that the differences in DNA content are due to differences in the amount of constitutive heterochromatin. On the other hand, analysis of DNA reassociation kinetics indicates that the differences in genome size per nucleus in these species are due to differences in the amount of highly repeated sequences and, to a greater extent, differences in the amount of moderately repeated sequences [48].

FISH with a telomeric probe reveals differences in the amount and localization of telomeric sequences in the analyzed species. Thus, while all the species show telomeric signal at the expected location at the ends of the chromosomes, only *E. calamita* has centromeric heterochromatic ITS located on all chromosomal pairs (not evident in subtelomeric pair 11). ITS have been reported in vertebrates [49], including bats [50], voles [51], turtles [52], fish [53] or amphibians [54]. The presence of ITS in pericentromeric regions could be a relic of structural chromosome rearrangements (e.g., chromosome fusions or pericentric inversions) [55]. It could be argued that the ITS from *E. calamita* were originated from chromosomal reorganizations (inversions and/or fusions), as has been proposed in the genus *Scarthyla* [56] or in Terranan frogs [29]. However, considering the conservation of the karyotypes in *B. spinosus*, *B. viridis* and *E. calamita*, with similar chromosome morphology and identical 2n (22) and FN (44) numbers, the presence of ITS in all centromeric positions in *E. calamita* would require reorganizations in all chromosomes of the karyotype. An alternative explanation for the existence ITS is that these sequences could have been inserted within unstable sites during the repair of DNA double-strand breaks (DSBs) [57], and then amplified by different mechanisms [55]. Finally, considering that the ITS in *E. calamita* are located in the centromeric heterochromatin of most chromosome pairs (not evident for pair 11), it is possible that the telomeric sequences are a component of the centromeric satellite DNA, amplified and extended to all centromeric positions. A similar explanation has been proposed (independent amplification of telomeric repeats) for the ITS described in some species of the genus *Phyllomedusa* [58].

Finally, we analyzed the composition and distribution of repetitive sequences by GISH. When chromosomes are hybridized with their own genomic DNA, intense signals are observed in centromeric positions in all species. Less intense signals can be identified in telomeric regions, but only when genomic DNA from *E. calamita* is used as probe. These terminal signals may be related with the presence of high amounts of centromeric ITS in this species, making the genomic DNA probe from this species highly enriched in telomeric sequences. Accordingly, the hybridization of *B. spinosus* and *B. viridis* chromosomes with a genomic probe from *E. calamita* reveal positive signals accumulated at the ends of the chromosomes.

Cross-GISH experiments show that the centromeric sequences are different in each species. This can be deduced by the loss of the centromeric signal when genomic DNA from other species (from different genera) were used as probes. It would be interesting to extend this analysis to other species from these genera to see if centromeric sequences are genus-specific, providing a useful tool for the taxonomic classification of bufonids.

## 5. Conclusions

Despite the advancement of massive DNA sequencing, conventional and molecular cytogenetic analyses are still relevant to study the genomic/chromosomal changes during evolution. Regarding the chromosomal diversification of these Bufonidae species, we have observed new undescribed interstitial C-bands, revealing population variability regarding heterochromatin distribution. The NOR shows size heteromorphisms, more frequent in *B. spinosus* and *E. calamita*. In *B. spinosus* the heteromorphism is not observed by FISH analysis using rDNA as probe, so it could be attributed to differences in activity between homologs. Regarding repeated sequences, *E. calamita* shows centromeric ITS located on all chromosomes, while the three species present accumulation of different repetitive sequences in centromeric regions. These chromosomal markers could contribute to shed some light into the taxonomic classification of bufonids. Finally, this manuscript provides the first detailed cytogenetic analysis available for *B. spinosus.*

## Figures and Tables

**Table 1:** Bufonidae samples included in this work.

Species	Origin	Ploidy	Sex	Dev. St.	Sample ID	Chr. Source	C-Band	Ag-NOR ^1^ (% HT)	FISH rDNA	TEL
*B. bufo*	Bologna (Italy)	2n = 22	F	A	H6	BM	-	-	-	7
*B. spinosus*	Jaén (Spain)	2n = 22	M	T	T8 (P5)	CC	-	14 (78.6%)	-	-
			F	T	T10 (P4)	CC	39	94 (71.3%)	-	-
			F	T	T12 (P2/P6/P9/P10)	CC	64	80 (88.7%)	5	18
*B. viridis*	Crete (Greece)	2n = 22	UK	T	R14 (P5/P6)	CC	68	55 (32.7%)	1	6
			UK	T	R15 (P4)	CC	-	-	4	11
*E. calamita*	Jaén (Spain)	2n = 22	UK	T	T2 (P21)	CC	3	162 (71.0%)	-	23
			F	T	T3.1 (P17)	CC	14	22 (72.7%)	-	-
			F	T	T3.2 (P10/P15/P16/P19)	CC	240	170 (74.1%)	4	69
			M	T	T4.4 (P18/P19)	CC	-	127 (81.9%)	-	-
			M	A	M4	BM	-	-	-	28

For each species the following information is provided: Origin: geographical origin of the samples; Ploidy: ploidy level; Sex (M: male; F: female; UK: unknown sex); Dev. St.: Developmental stage (A: adult; T: tadpole); Sample ID: identification of the sample—tadpoles were used to establish primary and secondary cell cultures, the passage number for the culture used to obtain metaphase chromosomes is indicated in parentheses; Chr. Source: Material used to obtain metaphase chromosomes (BM: bone marrow from adult animals; CC: cell culture from tadpoles). In each cytogenetic analysis (C-band, Ag-NOR, FISH with rDNA and TELomeric probes), the number of metaphases analyzed is indicated. ^1^ Percentage of heteromorphic (HT) NOR observed.

**Table 2:** Measures of chromosome lengths of pair 6 in *B. spinosus* Ag-stained metaphase plates from both sexes.

Sample ID	N	Sex	Lp(6pA-6pB)	Lq(6qA-6qB)	L(6A-6B)
T8	12	Male	7.477 ± 4.292	6.748 ± 6.325	5.462 ± 3.174
T10	18	Female	4.612 ± 4.105	5.452 ± 3.995	4.524 ± 2.579
T12	67	Female	5.883 ± 5.101	7.527 ± 5.104	5.168 ± 4.509

N is the number of metaphase plates analyzed. Lp(6pA-6pB), L(6A-6B) and Lq(6qA-6qB) are the mean of the differences between the relative lengths of homologues (A and B) for chromosome 6p, 6q and 6, respectively (mean ± SD in %).

## Data Availability

Not applicable.

## Supplementary Materials

The following supporting information can be downloaded at: https://www.mdpi.com/article/10.3390/genes13081475/s1, Figure S1: FISH for telomeric repeats in *E. calamita*; Figure S2: GISH in *E. calamita*.
