# Supplementary material for: Cytogenetic Analysis in the Toad Species Bufo spinosus, Bufotes viridis and Epidalea calamita (Anura, Bufonidae) from the Mediterranean Area
Source: Genes (Basel). 2022 Aug 18;13(8):1475. doi: 10.3390/genes13081475 (PMC9408106; doi:10.3390/genes13081475)
Supplement: Supplementary file 1 [file genes-13-01475-s001.zip › genes-1862403-supplementary.pdf]

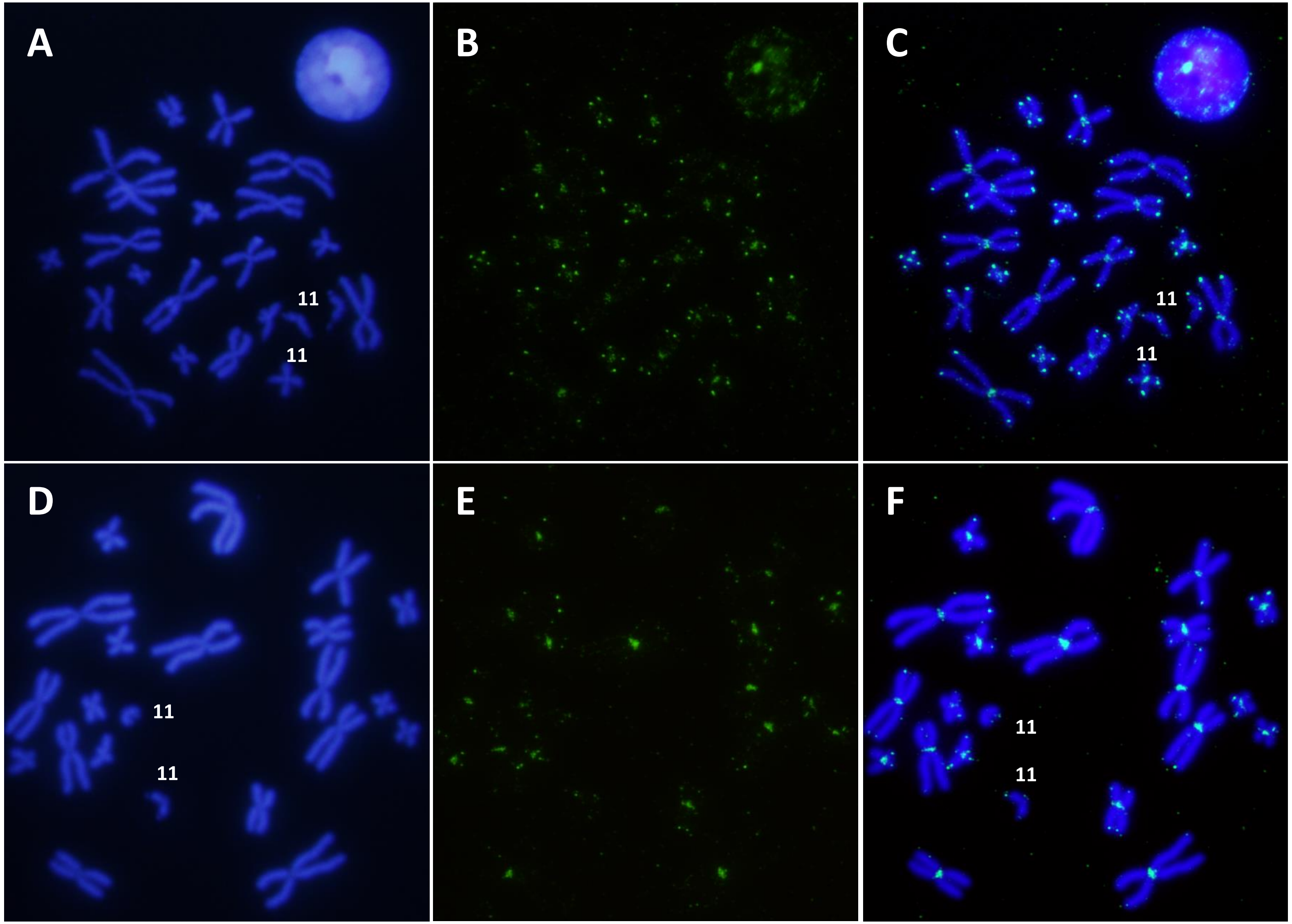

Supplementary Figure S1. FISH for telomeric repeats in *E. calamita*. Fluorescent *in situ* hybridization with a telomeric probe labeled with biotin 11–dUTP (after four rounds of amplification), on metaphase chromosomes from *E. calamita* adult (A, B and C) or tadpole (D, E, and F). A and D) DAPI; B and E) FITC; C and F) Merged. Pair 11 is easily identified by the presence of a secondary constriction on distal 11q. Supplementary Figure S1C corresponds to Figure 4D.

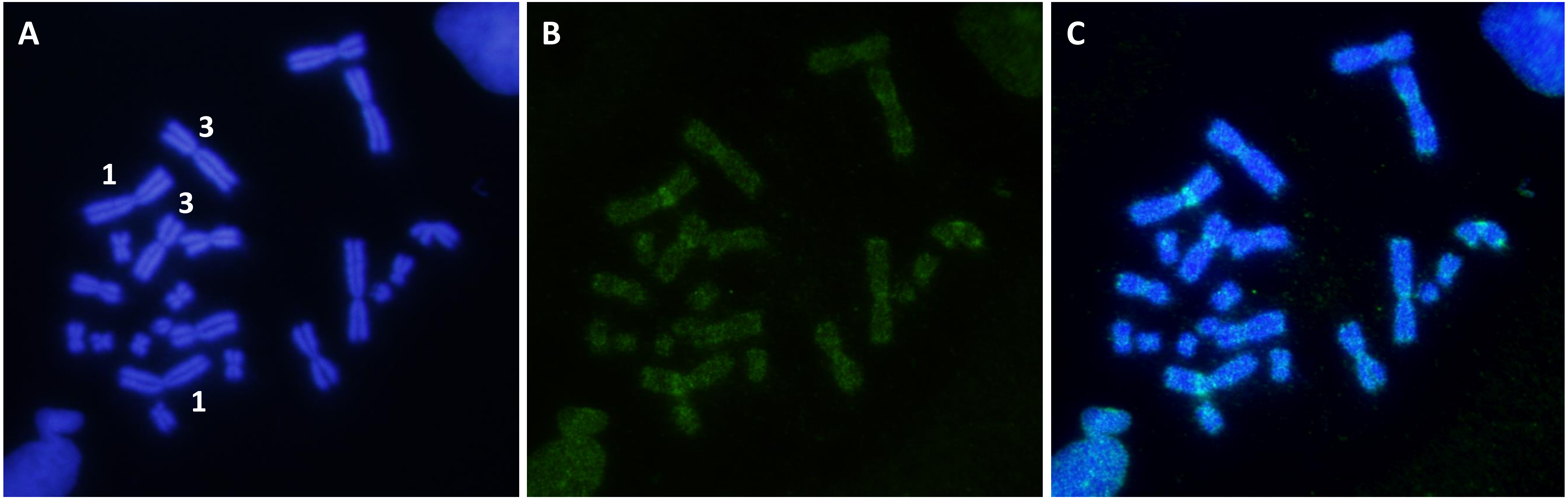

Supplementary Figure S2. GISH in *E. calamita*. Fluorescent *in situ* hybridization using genomic DNA (GISH) from *E. calamita* as probe, on metaphase chromosomes from *E. calamita*. The probe was labeled with biotin 11–dUTP and the results were obtained after three rounds of immunological amplification using an avidin-FITC/biotinylated anti-avidin system. A) DAPI; B) FITC; C) Merged.
